# Genetics of Frontotemporal Dementia in the Serbian Population: Findings from a Hospital-Based Cohort

**DOI:** 10.3390/neurolint17100162

**Published:** 2025-10-07

**Authors:** Vuk Milošević, Jelena Bašić, Marija Semnic, Eva Antić, Marina Malobabić, Milan Stoiljković

**Affiliations:** 1 Clinic of Neurology, University Clinical Center Niš, 18000 Niš, Serbiamarinasudimac@gmail.com (M.M.); 2 Department of Neurology, Faculty of Medicine, University of Niš, 18000 Niš, Serbia; 3 Department of Biochemistry, Faculty of Medicine, University of Niš, 18000 Niš, Serbia; jelena.basic@medfak.ni.ac.rs; 4 Clinic of Neurology, University Clinical Center of Vojvodina, 21000 Novi Sad, Serbia; marija.semnic@mf.uns.ac.rs; 5 Department of Neurology, Faculty of Medicine, University of Novi Sad, 21000 Novi Sad, Serbia; 6 Department of Pharmacology, Faculty of Medicine, University of Niš, 18000 Niš, Serbia; 7 Department of Comparative Medicine, Yale School of Medicine, Yale University, New Haven, CT 06510, USA

**Keywords:** frontotemporal dementia, FTLD, *MAPT*, *C9orf72*, *GRN*, Southeastern Europe

## Abstract

Background and objectives: Frontotemporal dementia (FTD) is a heterogeneous neurodegenerative disorder with autosomal dominant forms most often linked to *MAPT*, *GRN*, and *C9orf72*. We aimed to evaluate the prevalence of pathogenic variants in these genes in a hospital-based cohort of FTD patients assessed at a tertiary referral center in southeastern Serbia. Methods: We studied 58 consecutive patients with FTD spectrum syndromes evaluated at a tertiary referral center. All underwent standardized neurological, neuropsychological, and imaging assessments, and family history was recorded. Genetic testing included validated assays for *C9orf72* repeat expansions and next-generation sequencing of *MAPT* and *GRN*. Results: Women comprised 53.45% of the cohort. The mean age was 67.88 years, with mean onset at 61.70 years. Behavioral variant FTD predominated (75.87%), while language forms were less frequent. Positive family history was present in 16 patients (27.59%). Pathogenic variants were identified in three individuals (5.17%): two unrelated carriers of the intronic *MAPT* mutation c.1920+16C>T and one patient with a *C9orf72* expansion. No *GRN* variants were detected. Mutation frequency was 18.75% in familial cases, while none were found among sporadic patients (*p* = 0.018). Four of nine relatives were asymptomatic *MAPT* mutation carriers. Conclusions: This first genetic study of FTD in southeastern Serbia revealed a lower mutation frequency than in Northern and Western Europe, but similar to cohorts from Southeastern Europe. The detection of *MAPT* c.1920+16C>T in two unrelated families extends the geographic range of this splice-site variant and underscores the importance of systematic genetic testing and larger collaborative studies in the Balkans.

## 1. Introduction

Frontotemporal dementia (FTD) represents a spectrum of clinical syndromes caused by neurodegenerative diseases collectively referred to as frontotemporal lobar degeneration (FTLD). These entities are clinically, pathologically, and genetically highly heterogeneous. In a narrower sense, the clinical spectrum of frontotemporal dementias includes the behavioral variant of FTD (bvFTD), characterized by progressive changes in behavior and personality, as well as the primary progressive aphasias, most commonly the non-fluent/agrammatic variant (nfvPPA) and the semantic variant (svPPA), which predominantly affect language functions. In addition, although less frequent, either at disease onset or later in the course of progression, overlap with extrapyramidal disorders such as corticobasal syndrome (CBS) and progressive supranuclear palsy (PSP), or with motor neuron disease, may occur [1].

From a genetic perspective, most cases are sporadic; however, autosomal dominant mutations are registered in approximately 30–40% of cases [2]. The most common autosomal dominant familial forms of FTD are associated with mutations in three genes: *MAPT* (microtubule-associated protein tau), *GRN* (progranulin), and *C9orf72* (chromosome 9 open reading frame 72). These mutations result in distinct pathological changes and neurodegeneration, but ultimately lead to clinical syndromes with considerable phenotypic overlap [3].

The prevalence of different mutations varies across geographic regions and populations. For example, *C9orf72* repeat expansions show a markedly higher prevalence in Northern Europe, particularly in Scandinavia [4]. Likewise, *GRN* and *MAPT* mutations exhibit regional founder effects in certain European populations [5]. For southeastern Europe and the Balkan region, only a limited number of studies have examined the prevalence of these mutations. Available evidence suggests a potentially lower frequency of autosomal dominant mutations associated with the FTD spectrum compared to Northern and Western Europe [6,7]. The reasons for these regional differences may lie in population-specific genetic backgrounds, underdiagnosis, or limited access to genetic testing. Therefore, further research is necessary to provide a clearer picture of FTD genetics in southeastern Europe.

The aim of this study was to investigate the frequency of *MAPT*, *GRN*, and *C9orf72* mutations in a hospital-based cohort of patients evaluated at a tertiary university hospital serving the population of southeastern Serbia.

## 2. Materials and Methods

### 2.1. Study Population

**Figure 1:**
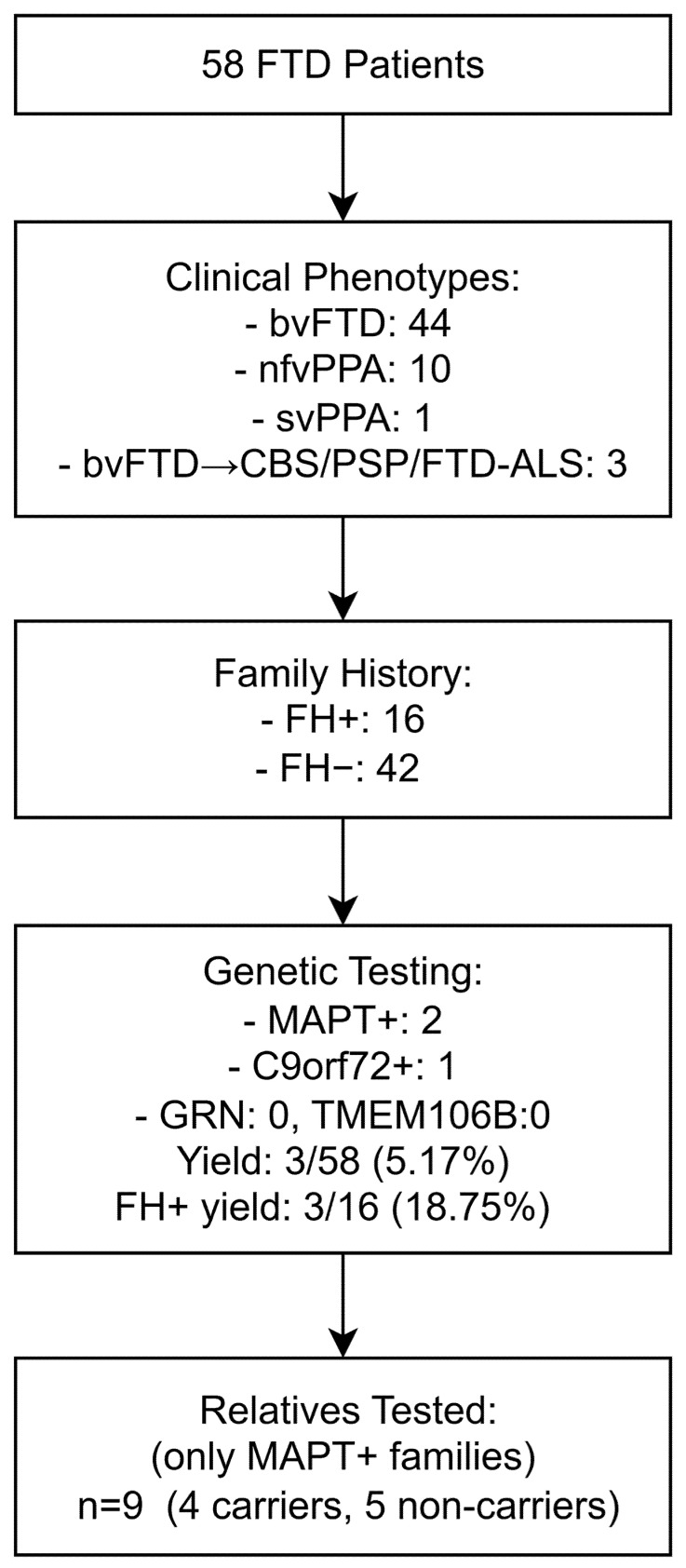
Flow chart of the study population, clinical classification, results of genetic testing of patients, and cascade testing of relatives. Note: MAPT+, patients positive for *MAPT* mutation; C9orf72+, patients positive for *C9orf72* expansion; FH+, positive family history; FH−, negative family history. Other abbreviations are listed at the end of the manuscript.

This study included 58 consecutive patients with a diagnosis of either bvFTD, nfvPPA, or svPPA who were evaluated at the Clinic of Neurology, University Clinical Center Niš, Serbia, between 2023 and 2025. This university hospital serves as a tertiary referral center for the southern and eastern regions of Serbia, covering a population of approximately 1.4 million people. The diagnoses of bvFTD, nfvPPA and svPPA were established according to internationally accepted criteria [8,9]. Patients with an initial presentation of bvFTD who subsequently developed extrapyramidal features consistent with CBS and PSP, or clinical features of amyotrophic lateral sclerosis (FTD-ALS), were also included [10,11,12]. This is a hospital-based, consecutive cohort from a tertiary referral center and does not represent a defined percentage of all FTD cases in the region served by the center. A flow chart of the study population and genetic testing is shown in Figure 1.

### 2.2. Clinical Assessment

Clinical data were obtained as part of the routine diagnostic work-up for patients with cognitive disorders. All patients underwent a standardized diagnostic protocol including neurological examination, cognitive screening, and behavioral evaluation, followed by laboratory investigations, brain computed tomography (CT) or magnetic resonance imaging (MRI), and detailed neuropsychological testing. For the purposes of this study, results from the Mini-Mental State Examination (MMSE) [13] and the Frontal Assessment Battery (FAB) [14] were extracted from the neuropsychological evaluation. In patients who provided informed consent, lumbar puncture with cerebrospinal fluid (CSF) biomarker analysis was performed to exclude Alzheimer’s disease (AD).

Family history was considered positive (FH+) if, based on information obtained from the patient, caregivers, family members, or available medical records, one or more relatives up to the third degree of kinship were identified with clinical syndromes consistent with FTLD spectrum disorders (bvFTD, PPA, FTD-ALS, ALS, CBS, PSP) or with undefined early-onset dementia, in accordance with Goldman categories 1–3 [15]; otherwise, patients were classified as FH−.

Genetic testing was performed for the three genes most commonly implicated in autosomal dominant forms of FTD: *MAPT*, *GRN*, and *C9orf72*. In addition, the *TMEM106B* gene, a known genetic risk modifier, was analyzed. Genetic counseling was offered to all patients, and genetic testing for the same panel of genes was made available to family members of identified mutation carriers.

### 2.3. Genetic Analysis

Genomic DNA was extracted from peripheral blood and analyzed at PreventionGenetics (Marshfield, WI, USA), a CLIA-certified (CLIA #52D2065132) and CAP-accredited (CAP #7185561) laboratory. The *C9orf72* hexanucleotide repeat expansion was evaluated with four complementary assays [16,17]. Two repeat-primed PCR assays targeted the 3′ and 5′ ends of the repeat, and two additional fragment-length assays provided size estimates for non-expanded alleles. This combination allows both detection of expanded alleles and sizing of shorter ones, up to ~40 repeats. Alleles with fewer than 25 repeats are considered normal, those with more than 30 as pathogenic, while the intermediate range (25–30 repeats) is regarded as uncertain. The method is reliable but not without its limitations: very large expansions cannot be sized, allelic dropout may occur in heterozygous cases, and variants outside the repeat region remain undetected.

For *GRN*, *MAPT*, and *TMEM106B*, sequencing was performed with a hybrid-capture next-generation sequencing (NGS) panel on a NovaSeq 6000 (Illumina, Inc., San Diego, CA, USA) platform using 150 bp paired-end reads. The assay covered all coding exons and plus 10 base pairs of flanking intronic sequences, as well as selected non-coding regions known to harbor pathogenic variants. Data processing relied on validated pipelines for alignment, variant calling, and quality checks. Average coverage was close to 100×, and more than 98% of bases were sequenced at a depth of at least 20×. Regions that fell below this threshold were resequenced by Sanger to ensure completeness.

Copy number variants (CNVs) for *GRN*, *MAPT*, and *TMEM106B* were evaluated from NGS data at PreventionGenetics using an internally developed read-depth algorithm that compares mean read depth and distribution for each target against multiple matched controls; neighboring target read depth and the zygosity of variants within each target were used to reinforce calls. The pipeline shows sensitivity approaching 100% for CNVs affecting ≥4 exons, whereas sensitivity for smaller events is lower (~75%). Where technically indicated, smaller CNVs were not reported if orthogonal confirmation was not feasible.

Primary processing (alignment, variant calling, and CNV calling) was performed at the sequencing laboratory (PreventionGenetics). The authors interpreted finalized clinical reports, which also included methodological details, and did not re-analyze raw data.

Variants were classified according to American College of Medical Genetics and Genomics and the Association for Molecular Pathology (ACMG/AMP) guidelines [18] into the standard categories: pathogenic, likely pathogenic, variant of uncertain significance (VUS), likely benign, benign, risk allele, or pseudodeficiency. Synonymous variants without predicted impact on splicing or protein function were generally considered likely benign and not reported. Similarly, benign, likely benign, risk, and pseudodeficiency variants were not included. Interpretation drew on both public and commercial databases, and nomenclature followed the recommendations of the Human Genome Variation Society (HGVS) (http://www.hgvs.org (accessed on 30 August 2025)).

### 2.4. Statistical Analysis

Statistical analyses were performed using SPSS Statistics (IBM Corp., version 31) and R (version 4.0.5). Categorical variables were summarized as absolute and relative frequencies with exact 95% confidence intervals calculated by the Clopper–Pearson method. Proportions were compared using Fisher’s exact test (two-sided). Risk differences were reported with 95% confidence intervals estimated by the Newcombe–Wilson score method (without continuity correction). Continuous variables were summarized as means ± standard deviations (SD) with ranges, and group comparisons (FH+ vs. FH−) were performed using the Mann–Whitney U test and the chi-square test, where appropriate. A *p*-value < 0.05 was considered statistically significant. Group-level statistical testing was performed only for FH+ vs. FH− patients. Findings from the mutation-carrier subgroup are shown descriptively.

### 2.5. Reporting Guidelines

This observational cohort study was conducted and is reported in accordance with the STROBE (Strengthening the Reporting of Observational Studies in Epidemiology) guidelines [19].

## 3. Results

A total of 67 individuals were included in the study, comprising 58 patients with FTD and 9 unaffected relatives from *MAPT*-positive families. Among the patients, 31 were female (53.45%) and 27 male (46.55%). The mean age was 67.88 ± 9.09 years (range 46–88), with a mean age at onset of 61.70 ± 8.71 years (range 42–75). The mean disease duration was 6.17 ± 3.31 years (range 2–16). Patients had on average 12.47 ± 2.98 years of education (range 4–21). Neuropsychological assessment showed moderate impairment, with a mean MMSE of 21.05 ± 6.95 (range 3–30) and a mean FAB of 8.58 ± 3.90 (range 2–17). The most common clinical phenotype was bvFTD, observed in 44 patients (75.87%), followed by nfvPPA in 10 (17.25%) and svPPA in 1 (1.72%). Three additional patients (1.72% each) later developed features of CBS, PSP, or FTD-ALS, although their initial presentation was characterized by behavioral changes consistent with bvFTD. This distribution reflects the expected phenotypic heterogeneity in tertiary-care FTD cohorts, with standardized diagnostic criteria applied across all cases. A positive family history (FH+) was documented in 16 patients (27.59%), while the remaining 42 patients were classified as FH− (Table 1).

Genetic analysis identified pathogenic variants in a minority of cases (Table 2). Two unrelated patients carried the heterozygous intronic *MAPT* pathogenic variant c.1920+16C>T, which affects splicing and is associated with autosomal dominant familial FTD. Both index cases presented with a bvFTD phenotype and had a positive family history. Genetic testing was extended to nine unaffected relatives from these two families. In Family A, two of four children were mutation carriers, whereas the proband’s mother and brother tested negative. In Family B, both children carried the *MAPT* variant, while the patient’s sister was non-carrier. Altogether, *MAPT* mutations were identified in six individuals (two affected patients and four unaffected relatives), corresponding to a carrier rate of 44.44% (4/9) among at-risk relatives. One additional patient with a bvFTD phenotype tested positive for a pathogenic *C9orf72* expansion (>30 repeats), while all other tested individuals carried normal alleles (<25 repeats). No intermediate alleles (25–30 repeats) were detected. No pathogenic variants were detected in *GRN* or *TMEM106B*, and no variants of uncertain significance (VUS) were identified. No exon-level CNVs were detected across *GRN*, *MAPT*, or *TMEM106B*. Analyses concerning the three mutation carriers are descriptive only (Table 3).

The overall diagnostic yield among clinically diagnosed FTD patients was 5.17% (3/58). Yield was higher among those with a positive family history (3/16, 18.75%) compared with those without (0/42, 0.00%; Fisher’s exact test, *p* = 0.018; risk difference 18.75%, 95% CI 3.98–43.01).

No significant demographic or clinical differences were observed between FH+ and FH− patients. Age at onset (U = 247.00, Z = −1.55, *p* = 0.121), disease duration (U = 324.50, Z = −0.20, *p* = 0.840), years of education (U = 281.00, Z = −1.00, *p* = 0.316), and sex distribution (χ^2^(1) = 0.276, *p* = 0.599) were similar across groups. MMSE (U = 233.50, Z = −1.79, *p* = 0.074) and FAB scores (U = 119.00, Z = −1.93, *p* = 0.054) tended to be lower in FH+ patients, but did not reach statistical significance.

## 4. Discussion

This study represents the first analysis of FTD genetics in a hospital-based tertiary care cohort from southeastern Serbia. Among 58 clinically diagnosed patients within the FTD spectrum, we identified pathogenic mutations in three individuals (5.2%), comprising two *MAPT* carriers and one *C9orf72* expansion carrier, while no *GRN* mutations were detected. Four unaffected relatives were also identified as *MAPT* mutation carriers. These findings may add to the evidence suggesting lower frequencies of pathogenic variants in the Balkans than in Northern and Western Europe, a notion supported by several other hospital-based cohorts across Europe.

In Northern Europe, particularly in Scandinavia, *C9orf72* has emerged as the predominant genetic cause of FTD. The initial Finnish observations [20] were later confirmed in Sweden, where mutations were found in 45 of 132 patients (34%). *C9orf72* accounted for more than a quarter of the entire cohort (26.5%), whereas *GRN* (6.8%) and *MAPT* (0.8%) were much less frequent. In Sweden, mutations were detected in 76% of patients with a strong family history but also in 20% of those classified as apparently sporadic, highlighting how differences in family history assessment can influence observed yields [21]. By contrast, in our series the overall yield was substantially lower (5.2%), with pathogenic variants confined to FH+ cases (18.8%), and none detected among sporadic patients.

In Central and Western Europe, somewhat different patterns have been observed. In a large German multicenter study [22], pathogenic variants were found in 18% overall and in 75% of FH+ cases, again dominated by *C9orf72* (51%), followed by *GRN* (28%) and *MAPT* (12%). In Belgium, 22% overall and 34% of FH+ cases carried a pathogenic variant, with *C9orf72* and *GRN* contributing equally (~10% each) and *MAPT* being less frequent (3%) [23]. In the UK, 19% of patients harbored mutations, with C9orf72 (35%) and *GRN* (33%) both common, but *MAPT* unusually prominent (29% of genetic cases) [24], a finding that may in part be related to a founder effect described in families of Welsh origin [25]. This latter observation is particularly relevant to our study, where *MAPT* mutation carriers were also identified.

Data from Southern and Southeastern Europe remain limited but are gradually emerging. In Southern Italy, Capozzo et al. examined 65 FTD patients (22 familial, 43 sporadic) and identified only one novel *GRN* splice-site mutation, corresponding to a very low overall yield of 1.5% (4.5% among familial cases), with no *C9orf72* or *MAPT* variants detected [26]. In Belgrade, Serbia, Stefanova et al. reported pathogenic variants in 7.8% of patients, mainly five *C9orf72* expansions and three *GRN* mutations, but no *MAPT* mutation carriers [6]. In Greece, Ramos et al. found a yield of 9.3%, with contributions from both *C9orf72* and *GRN* [27], while in a Bulgarian dementia cohort *C9orf72* expansions accounted for 3.7% of cases [7]. A study from Istanbul, Turkey, reported a higher overall yield of 17.8%, with pathogenic variants identified in *C9orf72*, *GRN*, and *MAPT* [28]. Although heterogeneous, these Southern and Southeastern European data consistently show lower yields than those reported in Northern, Central and Western Europe, where hospital-based series often range from 16–34%. Our present findings from Niš, Serbia (5.2% overall; 18.8% among FH+ patients) are in line with this pattern, slightly higher than Italy but lower than Turkey and Greece.

Within our series, 27.6% of patients had a positive family history. Although no significant demographic or clinical differences were observed between FH+ and FH− patients, the FH+ group tended to score lower on both MMSE and FAB. While not statistically significant, this pattern may hint at a more aggressive clinical course in familial cases, a possibility that deserves further study in larger cohorts.

To our knowledge, *MAPT*-positive cases had not previously been reported in Serbia. In the Serbian cohort from Belgrade, Stefanova et al. identified no *MAPT* mutation carriers [6]. Of particular interest in our series, therefore, is the detection of the *MAPT* c.1920+16C>T (also known as IVS10+16C>T) variant in two apparently unrelated families from southeastern Serbia. While *GRN* and *C9orf72* mutations are usually more frequent worldwide, *MAPT* variants remain relatively rare and often show geographic clustering. The intronic *MAPT* variant c.1920+16C>T shifts pre-mRNA splicing toward the inclusion of exon 10. Because exon 10 encodes one of the four microtubule-binding repeats of tau, its preferential inclusion increases the proportion of 4-repeat (4R) tau isoforms relative to 3-repeat (3R) tau [29]. This isoform imbalance promotes tau aggregation and represents a well-recognized pathogenic mechanism in FTLD-tau [30,31]. Together with the exonic variants c.837C>A (p.Asn279Lys, N279K) and c.902C>T (p.Pro301Leu, P301L), c.1920+16C>T is among the most frequently reported *MAPT* mutations, associated with a wide clinical spectrum including bvFTD, PSP-like, and occasionally AD-like presentations. [32,33]. A strong founder effect has been documented in North Wales, where genealogical and haplotype studies traced the origin of this variant to around 23 generations ago [25]. Against this background, the presence of the same mutation in two independent families from Serbia is noteworthy. It expands the known geographic distribution of *MAPT* c.1920+16C>T into the Balkans and underscores the importance of including *MAPT* in routine testing for the FTD spectrum in this region. Whether these families share a distant founder haplotype or represent independent mutational events remains an open question, one that could be clarified by future haplotype analyses.

This study has some limitations. The sample size is modest, and as a single-center hospital-based series it may not reflect the full spectrum of FTD genetics in the wider population of southeastern Serbia. We did not perform haplotype analyses, which prevents confirmation of a potential founder status. The small number of genetically confirmed cases also limits genotype–phenotype correlations, and the cognitive differences between FH+ and FH− groups, while suggestive, cannot be interpreted with confidence. As a single-center hospital-based series, the cohort may be subject to selection bias, and we were unable to quantify how representative it is of all regional FTD cases. Finally, as our testing panel focused on the three major genes, pathogenic variants in other FTD-associated genes may have been missed.

## 5. Conclusions

Despite these limitations, our study provides the first systematic data on FTD genetics from southeastern Serbia and adds to the emerging evidence of regional variation across Europe. We confirm that pathogenic variants account for only a subset of cases, at lower frequencies than in Northern and Western Europe, with an absence of *GRN* mutations in our cohort. The detection of the *MAPT* c.1920+16C>T mutation in two families illustrates how rare founder variants may also be present in the Balkans, highlighting the importance of systematic genetic testing in clinical practice. Larger collaborative studies, ideally including haplotype analysis, will be essential to clarify the origins of these variants and to improve genetic counseling, patient management, and research in the region.

## Figures and Tables

**Table 1:** Demographic and clinical characteristics of patients with frontotemporal dementia (*n* = 58).

Characteristic	Value
Number of patients	58
Sex (F/M)	31 (53.45%)/27 (46.55%)
Age (years)	67.88 ± 9.09 (range 46–88)
Age at onset (years)	61.70 ± 8.71 (range 42–75)
Disease duration (years)	6.17 ± 3.31 (range 2–16)
Years of education	12.47 ± 2.98 (range 4–21)
MMSE	21.05 ± 6.95 (range 3–30)
FAB	8.58 ± 3.90 (range 2–17)
Clinical phenotypes	bvFTD: 44 (75.87%) nfvPPA: 10 (17.25%) svPPA: 1 (1.72%) CBS: 1 (1.72%) PSP: 1 (1.72%) FTD-ALS: 1 (1.72%)
Positive family history	16 (27.59%)

Abbreviations: bvFTD, behavioral variant frontotemporal dementia; nfvPPA, non-fluent/agrammatic variant primary progressive aphasia; svPPA, semantic variant primary progressive aphasia; CBS, corticobasal syndrome; PSP, progressive supranuclear palsy; FTD-ALS, frontotemporal dementia with motor neuron disease; MMSE, Mini-Mental State Examination; FAB, Frontal Assessment Battery. Note: Patients classified under PSP, CBS, and FTD-ALS initially presented with clinical features fulfilling the diagnostic criteria for bvFTD.

**Table 2:** Genetic findings in patients with frontotemporal dementia (*n* = 58).

	Positive/Total Cases	% Positive	Exact 95% CI (Clopper–Pearson)
*MAPT*	2/58	3.45%	0.42–11.91%
*GRN*	0/58	0.00%	0.00–6.16%
*C9orf72*	1/58	1.72%	0.04–9.24%
*TMEM106B*	0/58	0.00%	0.00–6.16%
Diagnostic yield (total)	3/58	5.17%	1.08–14.38%
Diagnostic yield (FH+)	3/16	18.75%	4.05–45.65%

Abbreviations: *MAPT*, microtubule-associated protein tau; *GRN*, progranulin; *C9orf72*, chromosome 9 open reading frame 72; *TMEM106B*, transmembrane protein 106B; Note: *TMEM106B* is considered a genetic modifier rather than a causative gene; no pathogenic variants were detected in this cohort; FH+, positive family history.

**Table 3:** Clinical and genetic characteristics of mutation carriers.

Characteristic	Patient 1	Patient 2	Patient 3
Gene/Variant	*MAPT* c.1920+16C>T	*MAPT* c.1920+16C>T	*C9orf72* pathogenic expansion
Phenotype	bvFTD	bvFTD	bvFTD
Family history	Yes	Yes	Yes
Age at onset (y)	57	55	65
Disease duration (y)	7	13	5
Age (y)	64	68	70
MMSE	16	24	27
FAB	10	12	-
Education (y)	16	11	20
Sex	F	M	M

Abbreviations: bvFTD, behavioral variant frontotemporal dementia; MMSE, Mini-Mental State Examination; FAB, Frontal Assessment Battery. Note: Patient 1 belongs to Family A (6 relatives tested), Patient 2 belongs to Family B (3 relatives tested), and Patient 3 carries a *C9orf72* expansion; family members of Patient 3 were not tested.

## Data Availability

The data presented in this study were generated as part of the pre-screening procedure for the multicenter clinical trial DNLI-H-0001 (NCT05262023). Due to ethical and legal restrictions, including patient privacy and data protection regulations, the datasets are not publicly available. Anonymized data may be made available from the corresponding author upon reasonable request and with appropriate institutional and regulatory approvals.

## Abbreviations

The following abbreviations are used in this manuscript:

ACMG	American College of Medical Genetics and Genomics
AD	Alzheimer’s disease
ALS	Amyotrophic Lateral Sclerosis
bvFTD	Behavioral Variant Frontotemporal Dementia
CBS	Corticobasal Syndrome
CI	Confidence Interval
CLIA	Clinical Laboratory Improvement Amendments
CNV	Copy number variants
CSF	Cerebrospinal Fluid
CT	Computed Tomography
FAB	Frontal Assessment Battery
FH	Family History
FH+	Positive Family History
FH−	Negative Family History
FTD	Frontotemporal Dementia
FTD-ALS	Frontotemporal Dementia with Amyotrophic Lateral Sclerosis
FTLD	Frontotemporal Lobar Degeneration
GRN	Progranulin
HGVS	Human Genome Variation Society
MAPT	Microtubule-Associated Protein Tau
MMSE	Mini-Mental State Examination
MRI	Magnetic Resonance Imaging
nfvPPA	Non-Fluent/Agrammatic Variant Primary Progressive Aphasia
NGS	Next-Generation Sequencing
PPA	Primary Progressive Aphasia
PSP	Progressive Supranuclear Palsy
SD	Standard Deviation
SNV	Single Nucleotide Variant
svPPA	Semantic Variant Primary Progressive Aphasia
TMEM106B	Transmembrane Protein 106B
VUS	Variant of Uncertain Significance
